# Iodine Intake Estimated by 24 h Urine Collection in the Italian Adult Population: 2008–2012 Survey

**DOI:** 10.3390/nu13051529

**Published:** 2021-05-01

**Authors:** Roberto Iacone, Paola Iaccarino Idelson, Pietro Formisano, Ornella Russo, Cinzia Lo Noce, Chiara Donfrancesco, Paolo Emidio Macchia, Luigi Palmieri, Daniela Galeone, Andrea di Lenarda, Simona Giampaoli, Pasquale Strazzullo

**Affiliations:** 1 Department of Clinical Medicine and Surgery, Federico II University of Naples Medical School, 80131 Naples, Italy; ornella.russo@unina.it (O.R.); pmacchia@unina.it (P.E.M.); strazzul@unina.it (P.S.); 2 Department of Translational Medical Science, Federico II University of Naples Medical School, 80131 Naples, Italy; pietro.formisano@unina.it; 3 Department of Cardiovascular, Endocrine-Metabolic Diseases and Aging, Istituto Superiore di Sanità, 00161 Rome, Italy; cinzia.lonoce@iss.it (C.L.N.); chiara.donfrancesco@iss.it (C.D.); luigi.palmieri@iss.it (L.P.); simonagiampaoli@outlook.com (S.G.); 4 Center for Disease Prevention and Control, Italian Ministry of Health, 00161 Rome, Italy; d.galeone@sanita.it; 5 ANMCO, Italian Association of Hospital Cardiology, 50121 Florence, Italy; dilenar@units.it; 6 S.C. Cardiovascolare e Medicina dello Sport, Ospedale Maggiore di Trieste, 34122 Trieste, Italy

**Keywords:** iodine prophylaxis, iodine deficiency disorders, thyroid, adult age, iodine intake, 24 h urinary excretion

## Abstract

Monitoring the population iodine status is essential for iodine deficiency eradication. This study assessed the average dietary iodine intake and the iodine status of a random sample of the Italian general adult population. The study population included 2378 adults aged 35–79 years (1229 men and 1149 women) from all 20 Italian regions, participating in the Osservatorio Epidemiologico Cardiovascolare/Health Examination Survey 2008–2012 (OEC/HES), and were examined for iodine intake in the framework of the MINISAL-GIRCSI Programme. Dietary iodine intake was assessed by the measurement of 24 h urinary iodine excretion. The median daily iodine intake of the whole population was lower (96 µg/d, interquartile range 51–165) than the daily adequate iodine intake according to both EFSA and WHO recommendation (150 µg/d), with a significantly lower value among women (85 µg/d) compared with men (111 µg/d). Iodine intake diminished with age and increased with BMI (body mass index) in male but not in female participants, without achieving the adequate intake in any sex, age, or BMI category. In this random sample of Italian general adult population examined in 2008–2012, iodine intake still remained lower than the recommended values despite the implementation of a strategy of iodoprophylaxis based on salt iodization in 2005. These data represent a valuable reference for future monitoring of iodine status in our country.

## 1. Introduction

Iodine is an essential nutrient for mammals at all ages, being required for the synthesis of thyroid hormones, which play a fundamental role for a healthy growth and a normal neurological development in children [1] and for energy-yielding metabolic processes at all ages [2]. While in children, long-term inadequate iodine intake may cause insufficient cognitive development [3,4], in adults, iodine deficiency has been associated with various clinical disorders, such as goiter, increased risk of thyroid cancer, and clinical manifestations of hypothyroidism such as mental sluggishness, cold intolerance, and mixedema [5]. The definition of iodine adequate intake (AI) by the World Health Organization (WHO) was based on observational studies carried out in Central America and European school children, in which a low prevalence (less than 5%) of goiter was habitually observed in the areas characterized by an iodine concentration in the urines of 100 µg/L or higher [6]. Since approximately 92% of the ingested iodine is eliminated in the urines [7,8] and the average 24 h urine volume in adults is 1.5 L or more, WHO/FAO (Food and Agriculture Organization of the United Nations) set the AI for iodine in adults at 150 µg/d [9]. This value was confirmed in 2014 by an ad hoc EFSA panel of experts [8]. The average 24 h urinary iodine excretion is considered the best indicator of the habitual iodine intake of a given population. It is preferred to dietary assessment, which is felt to be much less accurate [10,11,12].

In many regions of the world, dietary iodine intake is not sufficient to meet the physiological requirements and to achieve an AI of the nutrient [13]. Following WHO advice, fortification of food-grade salt with iodine is used for the prevention of iodine deficiency disorders [14,15]. In Italy, the iodoprophylaxis strategy is regulated by the 55/2005 law, that requires the addition of potassium iodate to food grade salt in the amount of 30 mg per kilogram and the mandatory availability of iodized salt in food shops and supermarkets. Since 2009, responsibility for surveillance of the national program of iodoprophylaxis was given to the National Observatory for the Monitoring of Iodoprophylaxis (OSNAMI), established at the National Institute of Health. According to the Italian Ministry of Health, between 2011 and 2012 the percentages of iodized salt sold for household use and for the catering system were, respectively, 50 and 23% of the total sales of food grade salt [16]. An investigation carried out in 2013 among the Italian National Health Service General Practitioners and Pediatricians found that the recommendation to switch from the use of common salt to that of iodized salt was delivered to patients by less than 23% of physicians [17].

The aim of the present work was to evaluate the iodine intake level in the adult Italian population estimated by 24 h urine collection, between three to seven years after the introduction of the Italian legislation on iodoprophylaxis (55/2005), and to analyze the association between iodine intake and some anthropometric and demographic characteristics.

## 2. Materials and Methods

### 2.1. Study Population and Survey Protocol

Between 2008 and 2012, within the CUORE Project, the Italian National Institute of Health, in collaboration with the National Association of Hospital Cardiologists Heart Care Foundations (ANMCO-HCF), performed the Osservatorio Epidemiologico Cardiovascolare/Health Examination Survey (OEC/HES), in which 4344 men and 4370 women, aged 35–79 years, were examined [18]. The aim of the OEC/HES 2008–2012 was to describe the lifestyle, the risk factor profile, and the prevalence of high-risk conditions and of cardiovascular and chronic diseases in random samples of the general population, resident of 23 municipalities located in the 20 Italian regions, stratified by decades of age and sex. Mean participation rate to the survey was 53%. Participants self-reported socio-demographic information and lifestyle habits by a standardized questionnaire. Anthropometric measurements (weight, height, waist, and hip circumferences) were taken. Resting ECG, spirometry and bone densitometry were recorded, and the lipid profile was assayed. Participants were required to perform a 24 h urine collection according to detailed written instructions. The Italian National Institute of Health ethical committee approved the survey on 11 November 2009.

**Figure 1:**
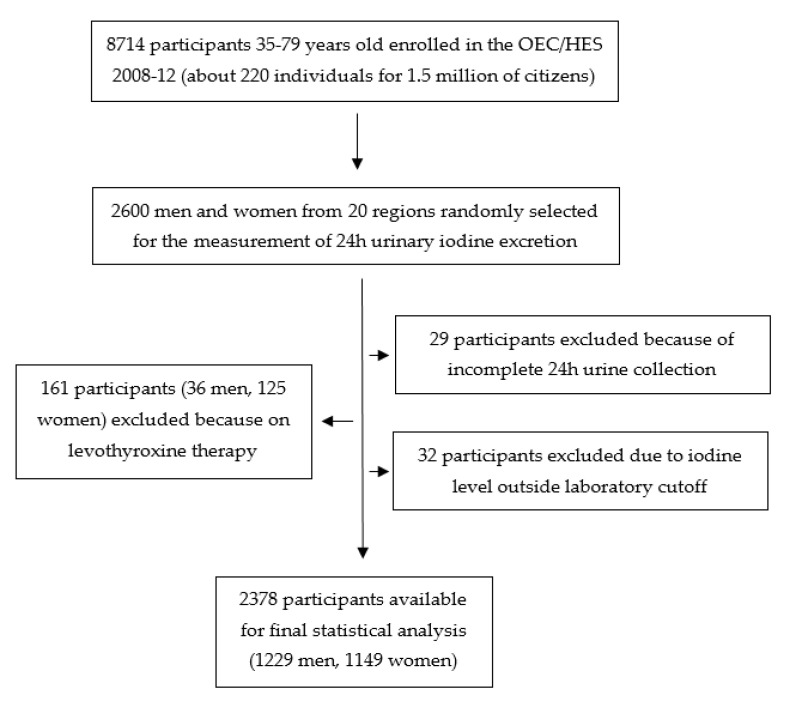
Flowchart of the study population.

To the purposes of the MINISAL-GIRCSI Programme, 2600 participants, representative of both sexes and all the Italian regions (about 130 participants per region) were randomly selected for the analysis of iodine intake. Twenty-nine subjects were excluded from the analysis because of suspected incomplete 24 h collection (participant report of incomplete urine collection, 24 h urine volume below 500 mL, or creatinine content referred to body weight lower than the mean minus two standard deviations from the population mean [19]). Twenty-four additional subjects were excluded because of a urinary iodine concentration (UIC) below the analytical sensitivity of the method (5 µg/L), and 8 subjects were excluded because their UIC was >400 µg/L, presumably due to occasional high iodine ingestion (food with very high iodine content, local antiseptic, disinfectants, or iodine-containing toothpaste). Finally, 165 participants (36 men and 125 women) were excluded from the analysis because of ongoing levothyroxine therapy. Detailed data about these participants are provided as supplementary material. Statistical analyses were performed on 2378 participants (1229 men and 1149 women), Figure 1.

### 2.2. Iodine Intake Assessment

After the clinical examination, each participant received a plastic container for the 24 h urine collection together with detailed instructions on how to perform urine collection. Briefly, participants had to discard the first void in the morning and collect all the urines until the first void of the following morning. Total 24 h urine volume was recorded. Three samples were aliquoted in plastic containers and immediately stored at −80 °C until the chemical analyses were performed. Creatinine and iodine measurements were carried out centrally, respectively, at the Departments of Clinical Medicine and Surgery and at the Department of Translational Medical Science, at Federico II University of Naples Medical School. Urinary iodine was analyzed as previously described [20] by an Autoanalyzer 3 system (Bran + Luebbe GmbH, Norderstedt, Germany), using the ceric-arsenious acid reaction and a modified digestion method, with the conventional acid digestion replaced by ultraviolet irradiation. Twenty-four-hour urinary iodine excretion (UIE) was expressed as micrograms per 24 h. Since 92% of the ingested iodine is excreted in urines [7,8], the daily iodine intake (DII) was estimated with the formula: DII (µg/d) = UIE/0.92

Urinary creatinine, measured by the kinetic Jaffé reaction using an ABX Pentra 400 apparatus (HORIBA ABX, Rome, Italy), was used as an indicator to assess the adequacy of the 24 h collection.

The prevalence of inadequate iodine intake in the study population was assessed with reference to the EFSA and WHO/FAO AI for iodine in adults [8] (150 µg/d as population median). The iodine status of the population was assessed according to WHO criteria, i.e., the population median UIC and the percentage of individuals with a concentration below 50 µg/L [15].

### 2.3. Statistical Analysis

Statistical analysis was performed using the Statistical Package for the Social Sciences (SPSS Statistics for Windows, Version 22.0; IBM Corp, Armonk, NY, USA). The descriptive statistics covered the whole study population and the population stratified by gender or by age and BMI classes. Since the numeric variables under investigation did not follow a Gaussian distribution (as assessed by Kolmogorov–Smirnov test), the results were expressed as median and interquartile range (IQR). For categorical variables, the results were reported as frequencies (%). Non-parametric tests were used to test for between-group differences (Mann–Whitney test in the case of two groups and the Kruskal–Wallis test for more than two groups). The Jonckheere–Terpstra test was used for the analysis of trend, and Spearman rank correlation analysis was used to assess the occurrence of statistical associations between the variables under investigation. Two-sided *p* values less than 0.05 were considered statistically significant.

## 3. Results

Table 1 shows the median and interquartile range (IQR) for age, weight, height, BMI, UIC, and DII of the whole study population and by gender. The mean ± SD of the DII for the whole study population was 127 ± 130 µg/d, UIC was 66 ± 68 µg/L, and 24 h urine volume was 1977 ± 586 mL.

Overall, 71.6% of participants had a DII below the suggested EFSA and WHO/FAO AI (150 µg/d). With reference to the WHO criteria for the assessment of the population iodine status, 80.2% of the participants had a UIC below 100 µg/L and 53.1% a UIC below 50 µg/L. Female participants had a lower body weight, height, and BMI and a lower daily intake of iodine as compared with male participants. Indeed, DII was below the AI in 78.7% of women and 64.9% of men (Chi-square = 55.2, *p* < 0.001); while UIC was below 100 µg/L in 86.2% of women and 74.5% of men (Chi-square = 51.3, *p* < 0.001). 

In the whole study population, DII was weakly and inversely associated with age (Spearman rho = −0.052, *p* = 0.011), and weakly and directly associated with BMI (Spearman rho = 0.085, *p* < 0.001). Similarly, UIC correlated weakly and inversely with age and weakly and directly with BMI (Spearman rho = −0.063, *p* = 0.002; Spearman rho = 0.088, *p* < 0.001, respectively). Separate correlation analysis by sex showed that associations of DII and UIC with, respectively, age and BMI were only present in men.

Upon stratification of the study population by age class (Table 2), a progressive decrease in iodine intake was apparent with increasing age in the whole population, with a significant negative trend for both UIC (*p* = 0.001) and DII (*p* = 0.009). The results were similar for male study participants, but not for female participants. Both with reference to the EFSA AI value for iodine intake and to the WHO standard for iodine status, the estimated median iodine intake was below the adequacy level in the whole population as well as in male and female participants in all age categories.

Table 3 reports the UIC and DII values by BMI class in the whole population and by gender. A progressive increase in iodine intake was apparent with increasing BMI in the whole population, with a significant positive trend for both UIC (*p* < 0.001) and DII (*p* < 0.001). A similar trend was observed in male but not in female participants. Median iodine intake was below the adequacy level in each BMI class with reference to both EFSA and WHO/FAO AI and WHO standard for iodine status, in the whole population as well as in male and female participants.

Table 4 shows medians and IQR of UIC and DII by Italian geographical area (Northern Italy, Central Italy, and Southern Italy) for the whole population and by gender. 

Northern Italy included eight regions (Piemonte, Lombardia, Veneto, Emilia-Romagna, Liguria, Valle D’Aosta, Trentino-Alto Adige, and Friuli-Venezia Giulia), Central Italy five regions (Toscana, Umbria, Lazio, Marche, and Sardegna), and Southern Italy seven regions (Campania, Basilicata, Puglia, Calabria, Sicilia, Molise, and Abruzzo). 

In the whole population and among male participants, significant differences were found both for UIC and DII when comparing the three geographical areas, where southern Italy had lower values. However, both with reference to the EFSA and WHO/FAO AI value for iodine and the WHO standard for iodine status, median iodine intake was below the adequacy level in the whole population as well as in male and female participants in all Italian geographical areas.

Concerning educational attainment, about half of the participants held a university degree or a high school certificate, without a significant difference by gender. On the other hand, a lower degree of educational attainment was more prevalent among women. A statistically significant trend in the daily iodine intake according to the educational attainment was detected in men but not in women, a lower iodine intake being observed among men with a lower educational level (Supplementary Figure S1).

Finally, Supplementary Table S1 shows that among the participants excluded from the main statistical analysis because they were on thyroid replacement therapy, the daily iodine intake estimated by 24 h urinary iodine excretion was on average 60 µg per day higher in men and 27 µg higher in women as compared with the rest of the study population, reflecting presumably a different mean dosage of the medication between men and women.

## 4. Discussion

This is, to our best knowledge, the only available report of a survey of iodine intake involving a random sample of adult general population recruited over the whole national territory with gold standard measurement based on 24 h urine collections. 

The main finding of the work is that, in a random sample of Italian adult population examined between 2008 and 2012, over 70% of the study population had an inadequate iodine intake according to both EFSA AI definition (150 µg/day) and WHO standard for iodine status (100–200 µg/L of urine). The iodine inadequacy was more severe in female compared to male participants, although the sex-related differences tended to reduce with increasing age. One possible explanation for this sex difference and for the age-related trend may be given by a larger consumption of food (and then a larger iodine intake from foods) and by a larger discretionary use of salt (and, thus, presumably also iodized salt) by men compared with women. In fact, iodine intake also increased with increasing BMI, in keeping with the reports of a greater salt intake [19] and with a possibly larger food consumption in overweight and obese individuals. These differences are larger in younger subjects and then attenuate later in life.

The data analysis by geographical area (North/Central/South) showed slight albeit significant regional differences: this notwithstanding, both UIC and DII values were below the WHO and EFSA recommended levels in every single geographical area.

The present results were obtained in a sample of adult general population examined between three and seven years after the introduction of the Italian legislation on iodoprophylaxis (55/2005). They are in keeping with the recently published results of another study carried out by our research group in a sample of the pediatric population in the same period. In this parallel study, involving a sample of 1270 healthy children and adolescents recruited in 10 Italian regions, approximately 50% of the female participants had a lower than adequate iodine intake [20]. Approximately, in the same period (2007–2012), an OSNAMI survey conducted on 7455 11–14 years school children residing in nine Italian regions showed that most of the regions were still iodine deficient having a median spot UIC less than 100 µg/L [16,21].

The iodine fortification of food grade salt was adopted in Italy well before the implementation of the 55/2005 law on iodoprophylaxis; however, the percentage sales of iodized salt were extremely low, and the prevalence of endemic goiter was high [22]. This prevalence has then gradually decreased despite the limited use of iodized salt possibly because of a spontaneous increase in iodine intake thanks to the increased consumption of iodine-rich foods associated with socioeconomic development [23].

The iodoprophylaxis campaign promoted by the Ministry of Health following the introduction of the legislation on iodoprophylaxis (55/2005) has made it possible to increase the percentage sales of iodized salt (for example, in the Abruzzo Region) from 13% in 1998 to 33.5% in 2001 and to further reduce the prevalence of goiter [16]. However, at the time of our survey (three to seven years after law 55/2005), the study population still had an inadequate iodine intake according to both the EFSA AI definition (150 µg/d) and WHO standard for iodine status (100–200 µg/L of urine).

The iodine status in Italy has also been investigated, using spot urine sampling, in some recent local studies relative to a few Italian regions. For example, in Veneto, a region in the north-east of Italy, a study carried out in 2018 and 2019 within the OSNAMI survey, on 747 school-age children with an average age of 13 years showed that in the entire cohort the median UIC was 111 µg/L (thus, within the range of adequacy set by WHO), yet 26% of the participants had a UIC below 50 µg/L [24]. The UIC was significantly higher among the iodized salt users than among non-users (117 versus 90 µg/L) as well as among regular consumers of cow’s milk compared with occasional consumers (132 vs. 96 µg/L): these results suggest that only the combined regular intake of cow’s milk and use of iodized salt allowed an adequate iodine status [24]. In Liguria, a region of north-west Italy, the median UIC values detected in adult subjects during the periods 2009–2013 and 2014–2018 were, respectively, 105 and 98 µg/L, indicating only a marginal iodine sufficiency in this region fourteen years after the enactment of law 55/2005 [25]. In this same population, the self-reported use of iodized salt was oddly low (between 13 and 29%) in the areas involved in the survey. In another recent study carried out in Calabria, a southern Italian region, a clear improvement in the population’s iodine status was observed after the promulgation of law 55/2005; however, areas of iodine deficiency were still present in the rural part of the region [26]. Additionally, in the study by Baldini et al. [27], carried out in Lazio, a region of Central Italy, a marked improvement was observed in the local population iodine status after the introduction of law 55/2005, suggesting a generally adequate iodine intake in school-age children. In the same study, however, two independent surveys of pregnant women showed that, despite the iodine sufficiency found in control women, most pregnant women and their fetuses were exposed to iodine deficiency. In an intervention trial evaluating the effects of iodine supplementation to pregnant women in Veneto, the median value of urinary iodine concentration at recruitment was only 56 µg/L, thus, well below the range of adequacy [28]. In the same region, a study of educational intervention in 970 school-aged children found a median value of urinary iodine concentration at baseline of only 70 µg/L, despite the use of iodized salt was reported by 78% of the participants [29]. Finally, a letter to the Journal of Endocrinological Investigation by OSNAMI investigators provided a preliminary short report of the results of a second survey conducted in the period 2015–2019 on 4000 school children aged 11–13 years residing in nine Italian regions showing overall a median UIC of 125 µg/L [30].

With regard to other European countries, Ittermann et al. have recently analyzed 40 studies from twenty-three countries and noted that iodine deficiency areas are still present in Europe [31]: in particular, standardized UIC values below 100 µg/L were reported in seven out of 13 studies available. According to the authors, the condition of persistent iodine inadequacy is most likely attributable to an insufficient use of iodized salt. In particular, in the study by Esche et al. [32], which was carried out in the same period of our study, the median DII of 6738 German adult subjects was lower than the EFSA and WHO/FAO AI. The authors estimated that 42% of the population total iodine intake was provided by the use of iodized salt and that this represented approximately 28% of the total salt used by the study participants. A more recent (2014–2017) UK survey in 2845 adults and children showed that the median UIC exceeded 100 µg/L only in regular consumers of cow milk, whereas the median UIC was lower in consumers of alternative types of milk [33]. Finally, the study of Trofimiuk-Müldner et al. on children of school age (*n* = 1000) and pregnant (*n* = 300) and breastfeeding women (*n* = 100) living in Poland showed that the mandatory sale of iodized salt to customers in the supermarkets markedly improved the iodine status in children but was not sufficient for pregnant and breastfeeding women [34].

A much better iodine status seems to be observed in China, according to a study by He et al. [35]. Here, the median DII of the studied population was as high as 165 µg/d. In China, the sale of iodized salt is mandatory, and, unlike Italy and other European countries, iodized salt is also regularly used by food manufacturers, accounting for up to 80% of the total salt intake.

Altogether, these findings suggest that the mere encouragement to use iodized salt at home and in the public restoration is not sufficient to reach a fully adequate iodine status in the whole population. The current Italian legislation on iodoprophylaxis delivered in 2005 aimed at the voluntary substitution of common salt with salt enriched with iodine (30 µg/g) by households, public restoration, and food industry. The law prescriptions, together with the educational campaigns conducted by public health authorities and scientific associations, brought about an overall improvement in the iodine status of the Italian population, compared with the previous situation of endemic iodine deficiency [36]. However, after the initial partial success of the prophylaxis campaign, further improvements have been lacking because of inherent limitations in the enforcement of the law and its prescriptions, which do provide for the mandatory availability of iodized salt in the large-scale distribution but leave the purchase and the use of non-iodized salt by consumers as well as by the catering system and the food industry substantially unrestricted. Indeed, according to a 2014 report by the National Institute of Health iodized salt represented only 54% of the total sales, 25% of the salt used by the catering system and a meagre 2–7% of the salt used by the food industry [16]. Even nowadays, most salty food products do not contain iodized salt. The major weaknesses of the current legislation hamper the possibility to further increase the population iodine intake, more so at a time when, thanks to the efforts of the same health institutions, the average total salt intake of the population tends to decrease [37]. This point is crucial, since non-discretionary salt intake, given by the salt added by the industry for food processing and storage (bread, pizza, cheese, ham and salami, sauces, snacks, etc.) represents at least 55% of the population total salt intake [38]. Considering that about 10% of the total salt intake is provided by the small amounts of salt contained in natural foods, only 35% of the total salt intake of Italian households is discretionary (salt added to foods while cooking or at the table). In an online survey of an opportunistic sample of Italian adult population (*n* = 11618) carried out in 2016–2018 [39], only 50% of the participants declared use of iodized salt on a regular basis, suggesting little change from previously published data in this regard [16]. Accordingly, the National Institute of Health Surveillance PASSI (Progress of the Health Authorities for Health in Italy) report indicated that, whereas 71% of a large sample of adult population declare the use of iodized salt, 53% stated to use it on a regular basis [40]. Thus, assuming that approximately 50% of the Italian adult population uses iodized salt at home on a regular basis, it may be estimated that 17.5% (50 × 0.35) of the total salt intake is given by iodized salt. Since at the time of our study, the average total salt intake of the Italian population was approximately 10 g/day [41], an absolute individual intake of 1.75 g of iodized salt may be estimated. As iodized salt in Italy contains by law 30 µg iodine per g of salt, which translates into 21 µg iodine per gram of ingested salt due to an estimated 30% loss during prolonged storage and cooking of food, iodized salt would have contributed on average only approximatively 37 µg (1.75 g × 21 µg/g) to the total daily iodine intake.

In 2014, a study by the Italian National Institute of Health [16] analyzed the average iodine content of different types of foods that are major sources of dietary iodine and matched the iodine food content with the median values of food consumption by the Italian population. The study led to estimate that an average amount of 60 µg/d of iodine was provided by food. Therefore, the expected total average iodine intake of the Italian population would have been approximately 97 µg/d (37 µg from iodized salt and 60 µg from food), a value which exactly matches the one detected in our study population by the measurement of 24 h urinary iodine excretion. Indeed, even if all the discretionary salt intake (about 3.5 g/day at the time of our study) was iodized, thus providing (3.5 × 21) 73.5 µg iodine/day, upon adding 60 µg coming from food, the total iodine daily intake would reach 133.5 µg/d, a value still below the EFSA/WHO adequate intake: this estimate stresses the need to substantially enhance the use of iodized salt by the food production system.

Major strengths of the current study are the use of a gold standard methodology for the assessment of total daily iodine intake, assayed centrally using 24h urine collections, the large size of our study population, and its coverage of the whole Italian territory (all twenty Italian regions). Careful recommendations were given to ensure proper urine collection, and the urines were checked for creatinine content to exclude from the analysis the individuals with possibly incomplete collections. Twenty-four-hour urinary iodine excretion is recognized as the best proxy for recent iodine intake [42,43] and is further amenable to be used to monitor the changes in habitual iodine intake occurring prospectively in a given population. Unfortunately, in epidemiological surveys, the collection of 24 h urine is not commonly used because it is felt bothersome to both the participants and the study investigators [44,45]. Most of the available studies assessed the iodine concentration (UIC) in spot urine samples. The UIC, although more practical, can be influenced by the hydration status of the subject and by the variation in iodine excretion throughout the day [12,46]. Thus, the measurement of 24 h urinary excretion provides a more accurate and reliable assessment of the iodine status. In our study, thanks to the availability of both UIC and UIE values, we were in the position to compare the differences observed using the two different measurement technics. Another strength of our study was the exclusion from the analysis of participants who were on hormone replacement therapy with levothyroxine, whose additional contribution to the subject iodine intake was on average 65 µg/d, a value similar to the average estimated amount of iodine provided by food.

A limitation of our study is that our study population was recruited almost 10 years ago. As a consequence, the results we reported depict a situation that is not updated. Nevertheless, as no further results have so far been reported from national surveys of the adult population, we believe that these results represent an essential reference for the assessment of ongoing and future changes in iodine status in Italy. Another limitation of the study is the lack of direct information about the use of iodized salt in the participants’ family environment. This information would have been useful to confirm the relationship between the regular use of iodized salt and a good iodine status. Finally, the use of a single 24 h urine collection to estimate the participants’ habitual iodine intake was also a limit: indeed, it is known that several 24 h collections are necessary to estimate the true “habitual” iodine intake of individuals with a high degree of accuracy [47]. For this reason, we refer in our report only to the median iodine intakes of the population as a whole or of population subgroups without attempting to describe the iodine intake or iodine status of the individual participants.

## 5. Conclusions and Perspectives

In summary, our study conducted between 2008 and 2012 on a large national sample of adult general population represents an important reference for future monitoring of iodine intake and of the population iodine status in Italy. Its findings confirm and integrate the results of another survey conducted in the same period and using similar gold standard methodology on a national sample of Italian pediatric population as part of the MINISAL-GIRCSI program. Both studies depicted a condition of iodine inadequacy relative to the time of the survey, also in agreement with a study conducted by the OSNAMI experts in the same period. More recent local studies as well as preliminary information about the results of a second OSNAMI survey conducted on school-age children seem to suggest a more favorable time trend, possibly related to the effects of the educational campaigns carried out by the health institutions and to the gradual implementation of the national strategy of iodoprophylaxis.

In the light of the data so far available and of the current trend to progressive salt intake reduction in many countries including our own [37], it appears all the more important to step up the efforts to enhance the regular use of iodized salt not only by households and by the public restoration system but also by the food industry and by the thousands of small enterprises of the food production system with particular regard to those involved in the manufacture of bread and other baking products known as a major source of salt intake in Italy and in several other countries. The possibility to make the fortification of animal feed with iodine more widespread in order to increase the iodine content of milk, dairy products, and meats also warrants careful consideration.

Finally, regular monitoring of the iodine intake of both the adult and children/adolescent population on the national territory, using gold standard methodologies, is strongly advisable.

## Figures and Tables

**Table 1:** Medians (IQR) of age, weight, height, BMI, urinary iodine concentration (UIC), 24 h urine volume, and daily iodine intake (DII), in the whole study population and by gender.

Basic Characteristics	Whole Population *n* = 2378	Men *n* = 1229 (51.7%)	Women *n* = 1149 (48.3%)	*p* *
Age, y	56 (46–67)	56 (45–66)	57 (46–67)	0.247
Weight, kg	73.0 (62.8–83.0)	79.2 (71.5–88.0)	64.4 (57.6–74.2)	<0.001
Height, cm	164 (157–171)	171.0 (165–176)	158 (153–162)	<0.001
BMI, Kg/m^2^	26.7 (24.0–30.2)	27.1 (24.9–30.2)	26.1 (23.0–30.2)	<0.001
UIC, µg/L	46 ** (23–88)	55 ** (27–101)	41 ** (20–72)	<0.001
Urine volume, mL/24 h	1900 (1500–2500)	1900 (1450–2500)	1950 (1500–2500)	0.156
DII, µg/d	96 *** (51–165)	111 *** (60–189)	85 *** (43–140)	<0.001

* Mann–Whitney U-test for independent samples (male vs. female participants). ** Median value below the WHO standard for adequate iodine status (100–200 µg/L). *** Median value below the EFSA and WHO/FAO daily adequate iodine intake (150 µg/day).

**Table 2:** Medians (IQR) of urinary iodine concentration (UIC) and daily iodine intake (DII) by age class in the whole study population and by gender.

Whole Population	35–44 y, *n* = 521	45–54 y, *n* = 570	55–64 y, *n* = 742	65–79 y, *n* = 545
UIC, µg/L	49 * (24–93)	51 * (27–95)	43 * (21–83)	43 * (20–82)
DII, µg/d	97 ** (52–160)	106 ** (60–176)	92 ** (49–164)	90 ** (44–152)
Male participants	35–44 y, *n* = 285	45–54 y, *n* = 291	55–64 y, *n* = 377	65–79 y, *n* = 276
UIC, µg/L	60 * (32–103)	63 * (33–114)	47 * (24–96)	48 * (20–92)
DII, µg/d	122 ** (65–192)	127 ** (75–196)	99 ** (56–183)	95 ** (45–179)
Female participants	35–44 y, *n* = 236	45–54 y, *n* = 279	55–64 y, *n* = 365	65–79 y, *n* = 269
UIC, µg/L	39 * (19–72)	43 * (24–73)	41 * (20–72)	39 * (19–70)
DII, µg/d	81 ** (39–128)	91 ** (49–146)	81 ** (41–144)	84 ** (41–135)

* Median value below the WHO standard for adequate iodine status (100–200 µg/L). ** Median value below the EFSA and WHO/FAO daily adequate iodine intake (150 µg/day).

**Table 3:** Medians (IQR) of urinary iodine concentration (UIC) and daily iodine intake (DII) by BMI class in the whole study population and by gender.

**Whole Population**	**BMI < 25 Kg/m^2^**	**BMI 25–29.99 Kg/m^2^**	**BMI ≥ 30 Kg/m^2^**
	***n* = 806**	***n* = 950**	***n* = 622**
UIC, µg/L	43 * (23–77)	46 * (22–87)	53 * (25–98)
DII, µg/d	90 ** (50–146)	96 ** (48–167)	106 ** (55–185)
**Male Participants**	**BMI < 25 Kg/m^2^**	**BMI 25–29.99 Kg/m^2^**	**BMI ≥ 30 Kg/m^2^**
	***n* = 329**	***n* = 575**	***n* = 325**
UIC, µg/L	49 * (25–89)	50 * (26–100)	68 * (30–110)
DII, µg/d	100 ** (55–172)	107 ** (60–188)	125 ** (65–211)
**Female Participants**	**BMI < 25 Kg/m^2^**	**BMI 25–29.99 Kg/m^2^**	**BMI ≥ 30 Kg/m^2^**
	***n* = 477**	***n* = 375**	***n* = 297**
UIC, µg/L	40 * (21–67)	39 * (18–71)	44 * (22–82)
DII, µg/d	84 ** (45–133)	87 ** (39–141)	83 ** (49–154)

* Median value below the WHO standard for adequate iodine status (100–200 µg/L). ** Median value below the EFSA and WHO/FAO daily adequate iodine intake (150 µg/day).

**Table 4:** Medians (IQR) of urine iodine concentration (UIC) and daily iodine intake (DII) by the Italian area and by gender.

**Whole Population**	**Northern Italy**	**Central Italy**	**Southern Italy**	*p* *
	***n* = 1150**	***n* = 515**	***n* = 713**	
UIC, µg/L	45 ** (24–84)	54 ** (26–91)	43 ** (20–89)	0.021
DII, µg/d	97 *** (55–158)	109 *** (55–179)	87 *** (44–165)	0.002
**Male Participants**	**Northern Italy**	**Central Italy**	**Southern Italy**	*p* *
	***n* = 585**	***n* = 281**	***n* = 363**	
UIC, µg/L	51 ** (28–97)	63 ** (31–114)	47 ** (24–101)	0.011
DII, µg/d	112 *** (62–185)	131 *** (63–205)	95 *** (52–181)	0.005
**Female Participants**	**Northern Italy**	**Central Italy**	**Southern Italy**	*p* *
	***n* = 565**	***n* = 234**	***n* = 350**	
UIC, µg/L	42 ** (22–71)	39 ** (21–70)	40 ** (18–74)	0.712
DII, µg/d	85 *** (48–141)	89 *** (47–139)	78 *** (38–137)	0.209

* Non-parametric Kruskal–Wallis independent test for three groups. ** Median value below the WHO standard for adequate iodine status (100–200 µg/L). *** Median value below the EFSA and WHO/FAO daily adequate iodine intake (150 µg/day).

## Data Availability

Not applicable.

## Supplementary Materials

The following are available online at https://www.mdpi.com/article/10.3390/nu13051529/s1, Figure S1: Median of iodine intake (µg/d) by educational attainment and gender, Table S1: Medians (IQR) of daily iodine intake (DII) in participants on levothyroxine therapy or not.
