# Supplementary material for: Iodine Intake Estimated by 24 h Urine Collection in the Italian Adult Population: 2008–2012 Survey
Source: Nutrients. 2021 May 1;13(5):1529. doi: 10.3390/nu13051529 (PMC8147380; doi:10.3390/nu13051529)
Supplement: Supplementary file 1 [file nutrients-13-01529-s001.zip › nutrients-1142237-supplementary.pdf]

Supplementary Figure 1

The supplementary figure 1 depicts the daily iodine intake values by sex and educational attainment. Data on educational attainment were available for 2236 participants. About half of the participants held a university degree (n = 378) or a high school certificate (n = 744), while 609 participants held a junior high school certificate, and 505 performed only the primary school. A lower degree of educational attainment was more prevalent among women. A statistically significant trend in the daily iodine intake according to the educational attainment was detected in men but not in women, a lower iodine intake being observed among men with a lower educational level (p = 0.016, Jonckheere–Terpstra test for p trend).

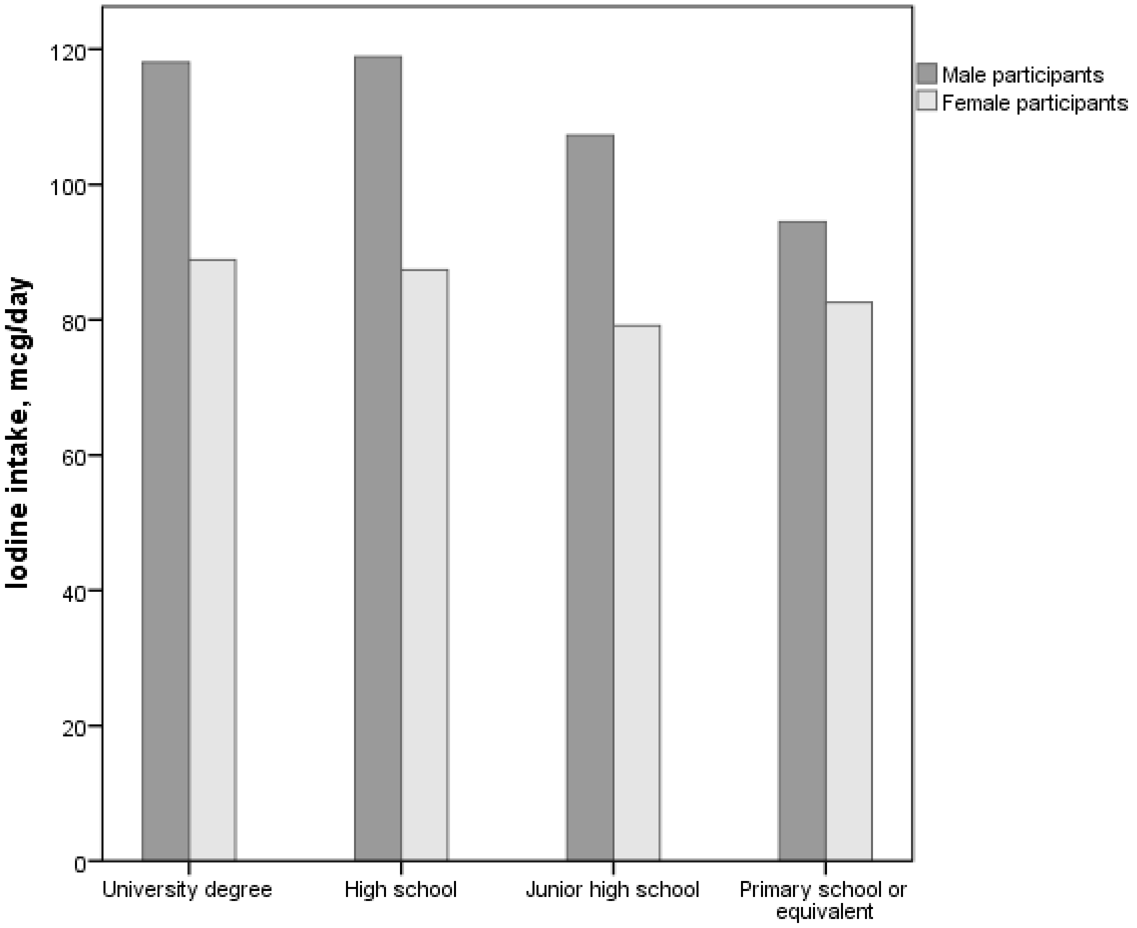

Figure S1. Median of iodine intake (μg/d) by sex and educational attainment.

Supplementary Table 1

The supplementary table 1 shows the daily iodine intake values in participants who were or were not on levothyroxine treatment. Among the 161 study participants on levothyroxine, 36 were male and 125 female. The average dose of levothyroxine was 110 μg/d in men and 90 μg/d in women. Sixty eight percent of the male participants took a levothyroxine dose between 100 and 150 μg/d, the remainderwere on a lower dose. In the female study participants, 73% were on levothyroxine 50–100 μg/d, 24% on a dose above 100 and only 3% on a dose below 50 μg/d.

Table S1. Medians (IQR) of daily iodine intake (DII) in participants on levothyroxine therapy or not.

**Whole population**	**On therapy** **n = 161**	**Not in therapy** **n = 2378**
DII, μg/d	125 (72–191)	96 (51–165)
Male participants	On therapy n = 36	Not in therapy n = 1229
DII, μg/d	171 (95–283)	111 (60–189)
Female participants	On therapy n = 125	Not in therapy n = 1149
DII, μg/d	112 (69–171)	85 (43–140)

The difference in iodine intake between patients following or not levothyroxine therapy could be explained by considering that the complete metabolism of levothyroxine provides approximately 65 μg of iodine per 100 μg of the drug.
